# Phase II study of adjuvant chemotherapy with pemetrexed and cisplatin with a short hydration method for completely resected nonsquamous non‐small cell lung cancer

**DOI:** 10.1111/1759-7714.13567

**Published:** 2020-07-30

**Authors:** Motoko Tachihara, Ryota Dokuni, Keiko Okuno, Shuntaro Tokunaga, Kyosuke Nakata, Naoko Katsurada, Masatsugu Yamamoto, Tatsuya Nagano, Kazuyuki Kobayashi, Yugo Tanaka, Yasuhiro Funada, Yoshimasa Maniwa, Yoshihiro Nishimura

**Affiliations:** ^1^ Division of Respiratory Medicine, Department of Internal Medicine Kobe University Graduate School of Medicine Kobe Japan; ^2^ Department of Respiratory Medicine Aijinkai Takatsuki General Hospital Takatsuki Japan; ^3^ Division of Thoracic Surgery Kobe University Graduate School of Medicine Kobe Japan

**Keywords:** Adjuvant chemotherapy, cisplatin, non‐small cell carcinoma, pemetrexed, short hydration

## Abstract

**Background:**

Cisplatin (CDDP) and vinorelbine as an adjuvant chemotherapy improve the overall survival of patients with completely resected non‐small cell lung cancer (NSCLC). However, the treatment completion rate is low due to severe adverse events (AEs). Pemetrexed (PEM) has been used in advanced NSCLC due to its high safety and efficacy. Additionally, the safety of a short hydration method for CDDP administration has been previously reported. Here, we investigated the feasibility of CDDP plus PEM with a short hydration method as adjuvant chemotherapy.

**Methods:**

A total of 21 completely resected nonsquamous NSCLC patients with pathological stage IIA to IIIA disease were enrolled into the study. Adjuvant chemotherapy consisted of four cycles of CDDP (75 mg/m^2^) plus PEM (500 mg/m^2^) every three weeks with a short hydration method. The primary endpoint was the treatment completion rate, and the secondary endpoints included toxicity, the two‐year relapse‐free survival (RFS) rate, and the outpatient treatment rate.

**Results:**

A total of 21 patients (median age: 66 years; 12 males) were enrolled in two centers. All cases were adenocarcinoma with PS0 (71.4%) or PS1 (28.6%). A total of 81.0% of the patients received four cycles of therapy as scheduled and the primary endpoint was met. The rate of outpatient chemotherapy completion after the second cycle was 90.5%. The grade 3 or higher toxicities were anorexia (*n* = 2) and pulmonary thromboembolism (*n* = 1). No grade 3/4 hematological toxicities or creatinine level elevations were observed. The two‐year RFS rate was 57.3%.

**Conclusions:**

CDDP and PEM with a short hydration is well tolerated in the outpatient setting with limited toxicity.

**Key points:**

**Significant findings of the study:**

CDDP plus PEM adjuvant therapy with a short hydration method is well tolerated in the outpatient setting with limited toxicity.

**What this study adds:**

CDDP plus PEM with a short hydration method has the potential to be one of the options of adjuvant therapy in the future.

## Graphical abstract

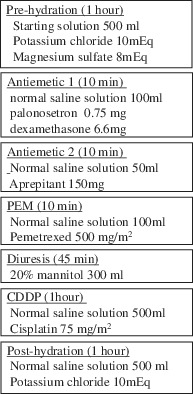

## Introduction

The number of cancer patients globally has increased 28% in the last decade, and the leading cause of cancer‐related death is lung cancer.^1^ Non‐small cell lung cancer (NSCLC) accounts for over 80% of all lung cancers. Even in stage I to III NSCLC, where surgical resection is indicated as the first‐line of treatment, the five‐year postoperative survival rates are reported to be 64.1% for stage IIA, 56.1% for stage IIB, and 47.9% for stage IIIA, which are not satisfactory.^2^ Cisplatin (CDDP)‐based adjuvant chemotherapy improves the five‐year survival and relapse‐free survival (RFS) of patients with completely resected stage II or III NSCLC.^3^, ^4^, ^5^, ^6^ CDDP‐based adjuvant chemotherapy is a standard postoperative therapy worldwide.

The main agent combined with adjuvant CDDP‐based chemotherapy is vinorelbine (VNR). However, the problems are discontinuation of adjuvant CDDP plus VNR treatment in approximately 50% of patients, treatment delay in 55%, and dose reduction in 77% due to adverse events (AEs) and patient rejection.^3^, ^4^, ^5^, ^7^ In addition, patients receiving cisplatin are conventionally required to be hospitalized and treated with a large amount of hydration to avoid nephrotoxicity. This may be one of the causes of impairing patients' quality of life (QOL). Therefore, reducing toxicity to improve dose delivery and establishing an outpatient‐based cisplatin administration regimen remain unmet needs for adjuvant treatment of NSCLC.

In recent years, CDDP and pemetrexed (PEM) have been widely used in advanced nonsquamous NSCLC due to their high safety and efficacy compared with those of other platinum combinations.^8^, ^9^, ^10^ In addition, the tolerance of a small amount of hydration, so called, a short‐term hydration method, has been shown by using appropriate replacement infusion,^11^, ^12^, ^13^ since renal dysfunction induced by CDDP occurs within four hours. However, it is unknown whether short hydration of CDDP is safe for patients with lobectomy.

Thus, we hypothesized that postoperative chemotherapy with a CDDP and PEM regimen with a short hydration method would have a better completion rate than conventional full‐hydration CDDP‐based chemotherapy. We conducted the first prospective multicenter phase II trial to evaluate the feasibility and efficacy of a CDDP and PEM regimen with a short hydration method for completely resected stage II and III NSCLC.

## Methods

### Patient selection

Eligible patients were aged 20–75‐years‐old with completely resected and pathologically confirmed stage II to IIIA nonsquamous NSCLC (according to the seventh edition of the Union for International Cancer Control Manual of Clinical Oncology TNM staging system). Additional eligibility criteria were an Eastern Cooperative Oncology Group performance status score of 0–1, adequate organ function (neutrophil count ≥1500/mm^3^, platelets ≥100 000/mm^3^, hemoglobin ≥9.0 g/dL, serum bilirubin ≤1.5 mg/dL, aspartate aminotransferase and alanine aminotransferase ≤2.5 times the upper limit of the normal [ULN] range, creatinine ≤1.2 mg/dL, creatinine clearance ≥60 mL/minute, and arterial oxygen pressure ≥ 60 mmHg or percutaneous oxygen saturation concentration [SpO_2_] ≥90%).

This multicenter, single‐arm phase II study was approved by the institutional review board of each institution and conducted in accordance with the Helsinki declaration of the World Medical Association. This study has been registered under the University Medical Hospital Information Network in Japan (UMIN000010336). All patients provided written informed consent before enrollment.

### Treatment

Eligible patients received adjuvant chemotherapy with four cycles of CDDP (75 mg/m^2^) plus PEM (500 mg/m^2^) with vitamin supplementation every three weeks with a short hydration method within eight weeks after surgery. The first cycle was administered with hospitalization, and the others were administered in the outpatient clinic in principle. The doses of CDDP and PEM were reduced one step (60 mg/m^2^ CDDP and 400 mg/m^2^ PEM) if any of the following toxicities occurred: grade 4 neutropenia or thrombocytopenia; grade 3 or higher febrile neutropenia; and grade 3 or higher nonhematological toxicities. The protocol treatment was discontinued if the patients had recurrence or any of the following events occurred: three weeks or longer treatment interruption; requirement for further dose reduction; grade 4 or higher nonhematological toxicities; patient's condition worsened due to adverse event; and patient refusal.

**Figure 1:**
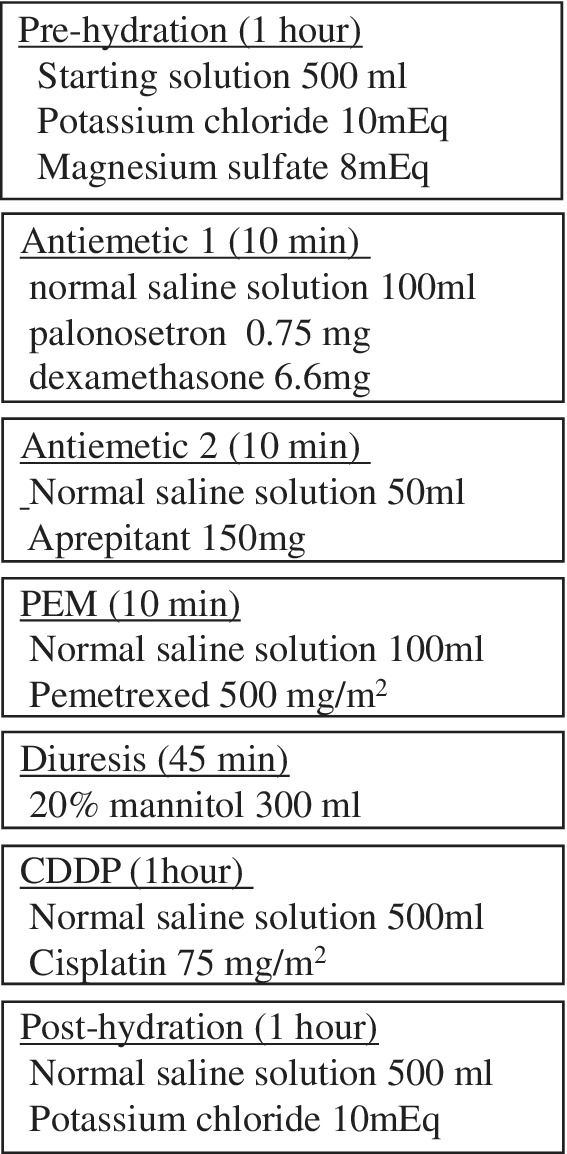
Detail of cisplatin (CDDP) and pemetrexed (PEM) short hydration regimen.

After antiemetic premedication (palonosetron, aprepitant, and dexamethasone) administration, PEM was administered. CDDP dissolved in 500 mL of normal saline solution was administered in an hour long infusion. Before and after CDDP administration, prehydration (potassium chloride and magnesium sulfate dissolved in 500 mL of starting solution) and posthydration (potassium chloride in 500 mL of normal saline) solutions were each infused for one hour. Patients received 300 mL of 20% mannitol by infusion over 45 minutes just before CDDP administration (Fig 1). All the patients drank more than 1000 mL of water daily on days 1 to 3 and took 8 mg of dexamethasone orally on days 2 and 3.

### Assessment

The primary endpoint was to evaluate the completion rate of four cycles of CDDP plus PEM as an adjuvant chemotherapy with a short hydration method for resected NSCLC patients. The secondary endpoints were used to evaluate safety, the outpatient treatment rate (rate of outpatient chemotherapy completion at the second cycle), and the two‐year relapse‐free survival (RFS). Physical examination parameters, complete blood cell counts and biochemical parameters were assessed at every cycle. Adverse events were evaluated according to the National Cancer Institute‐Common Toxicity Criteria for Adverse Events (CTCAE), version 4.0. Patients self‐reported their condition on days 1–7 of every cycle after administration on a checklist that included factors such as body temperature, bodyweight, nausea or vomiting, amounts of meal intake and drinking water intake, and urination frequency.

After completion or discontinuation of the protocol treatment, all patients were followed by physical examination, chest X‐ray every three months, and laboratory tests. Chest and abdominal computed tomography (CT) was performed every six months. If any signs of recurrence were observed, necessary examinations were added. Two years after registration, full staging (chest and abdominal CT, brain magnetic response imaging [MRI] or brain CT, and 18F‐FDG PET) was performed. All patients were followed until disease relapse or the cutoff date (March 2018).

### Statistical analysis

The study was designed as a prospective, single‐arm, multicenter phase II study. The sample size was calculated with an expected completion rate of 80% and a lowest limit of interest of 50% with an alpha value of 0.05 (two‐sided) and a detection power of 80%. The minimum sample size was 19. Considering a 10% rate for exclusion or deviation, the sample size was set at 21. RFS was analyzed using the Kaplan‐Meier method to estimate the median with a 95% confidential interval (CI). All statistical analyses were performed using EZR (Saitama Medical Center, Jichi Medical University, Saitama, Japan, version 1.38), which is a graphical user interface for R (The R Foundation for Statistical Computing, Vienna, Austria, version 3.3.2).^14^

## Results

### Patient characteristics

A total of 21 patients were enrolled between April 2013 and May 2017 from two institutions in Japan. Patient characteristics are shown in Table 1. All of the patients were assigned to the intervention group, and no patients were excluded or deviated. The median age was 66 years (ranging from 57 to 75 years), and the patient cohort comprised 12 (57.1%) men and nine (42.9%) women. All cases were adenocarcinoma with PS0 (71.4%) or PS1 (28.6%). There were 11 patients (52.4%) at pathological stage IIA, four (19.0%) at stage IIB, and six (28.6%) at stage IIIA.

**Table 1:** Patient characteristics

Characteristics		No. of patients (*n* = 21, %)
Age, median (range)		66 (57–75)
Sex	Male	12 (57.1)
	Female	9 (42.9)
ECOG performance status	0	15 (71.4)
	1	6 (28.6)
Histology	Adenocarcinoma	21 (100)
pStage	IIA	11 (52.4)
	IIB	4 (19.0)
	IIIA	6 (28.6)
Surgical procedures	Lobectomy	20 (95.2)
	Segmentectomy	1 (4.8)
Serum creatinine (mean ± SD, mg/dL)		0.70 ± 0.12
EGFR mutation	Positive	4 (19.0)
	Wild	12 (57.1)
	Unknown	5 (23.8)

### Treatment delivery

A total of 17 patients completed the four cycles of protocol chemotherapy, representing a completion rate of 81.0% (95% CI: 58.1–94.6) (Table 2). The rate of outpatient chemotherapy completion after the second cycle was 90.5%. Only one patient was unable to undergo outpatient treatment because grade 3 anorexia had occurred in the first course and the patient requested subsequent treatment to take place in hospital. A total of 17 patients (80%) received chemotherapy as scheduled. The chemotherapy protocol was discontinued in three patients because they developed pulmonary thromboembolism (*n* = 1), pneumonitis (*n* = 1), or anorexia (*n* = 1). One patient needed to delay the third cycle of chemotherapy because of neutropenia. One patient needed a one‐step dose reduction due to anorexia.

**Table 2:** Treatment delivery

Delivery status	No. of patients (%)
Patients completed cycles	
Cycle1	21 (100%)
Cycle 2	20 (95.2%)
Cycle 3	18 (85.7%)
Cycle 4	17 (81.0%)
Outpatient administration at second cycle	19 (90.5%)
Dose reduction of chemotherapy	1 (4.8%)
Delayed administration at least once	1 (4.8%)

### Safety

The treatment‐related adverse events that occurred are summarized in Table 3. The grade 3 or higher toxicities were anorexia (*n* = 2) and pulmonary thromboembolism (*n* = 1). No grade 3/4 hematological toxicities or creatinine level elevations were observed. No treatment‐related deaths occurred.

**Table 3:** Common treatment‐related adverse events (AEs)

	Grade 1	Grade 2	Grade 3	All grade
	n (%)	n (%)	n (%)	n (%)
Neutropenia	2 (9.5)	4 (19.0)	0	6 (28.6)
Anemia	15 (71.4)	0	0	15 (71.4)
Thrombocytopenia	3 (14.3)	0	0	3 (14.3)
Febrile neutropenia	—	—	0	0
Increased AST	2 (9.5)	0	0	2 (9.5)
Increased ALT	4 (19.0)	0	0	4 (19.0)
Increased creatinine	1 (4.8)	0	0	1 (4.8)
Anorexia	9 (42.9)	4 (19.0)	2 (9.5)	15 (71.4)
Vomiting	5 (23.8)	0	0	5 (23.8)
Diarrhea	1(4.8)	0	0	1 (4.8)
Pneumonitis	1(4.8)	0	0	1 (4.8)
Gingival pain	2 (9.5)	0	0	2 (9.5)
Rash	1 (4.8)	0	0	1 (4.8)
Thromboembolism	0	0	1 (4.8)	1 (4.8)
Infection without neutropenia	0	1 (4.8)	0	1 (4.8)

### Efficacy

**Figure 2:**
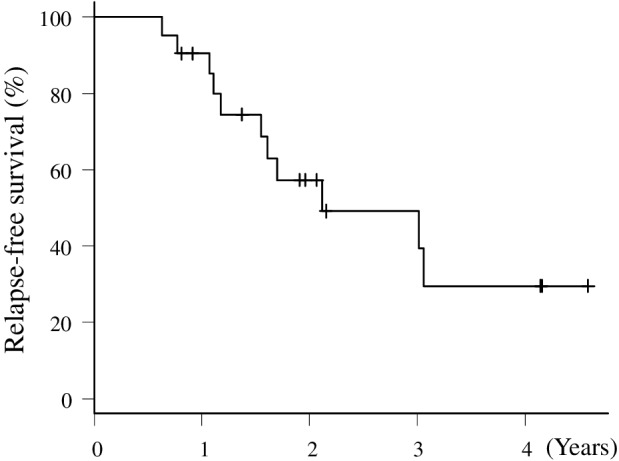
Kaplan‐Meier relapse‐free survival (RFS) curve of the patients.

All patients were followed and evaluated for relapse. The median follow‐up time was 20.7 months (range, 7.6–55.9 months). The median time to relapse was 25.8 months (95% CI: 19.6–NA), and the two‐year RFS rate was 57.3% (95% CI: 32.2–76.1) (Fig 2).

## Discussion

The present study was a prospective trial to investigate the feasibility of CDDP plus PEM adjuvant chemotherapy with a short hydration method for patients with resected nonsquamous NSCLC. The completion rate for CDDP plus PEM exceeded the assumed lower limit and represented a high completion rate over 80%. In addition, treatment delay and dose reduction were seen in only one patient (4.8%) in the present study, whereas these events have been reported to occur in 55% and 77% of patients receiving the CDDP plus VNR regimen, respectively.^3^, ^4^, ^5^, ^7^ The CDDP and VNR regimen is reported to have severe hematological toxicities; neutropenia ≥ grade 4 is observed in 55% of patients, infection ≥ grade 3 is observed in 8%, and febrile neutropenia is observed in 9%. In the present study, CDDP and PEM with a short hydration regimen resulted in no severe hematological toxicities or creatinine level elevations and only relatively mild vomiting and anorexia. Consequently, 80% of patients completed their treatment, which ensured the feasibility of this adjuvant chemotherapy regimen. Factors such as weight, nausea, and food intake in the self‐reported checklist also showed that this regimen was well tolerated.

Kreuter *et al*. performed a randomized phase II trial of NSCLC adjuvant chemotherapy with CDDP and PEM versus CDDP and VNR.^15^ They reported that the feasibility rates were significantly higher in the PEM arm (95.5% vs. 75.4%). Recently, Kenmotsu *et al*. presented a randomized phase III trial of NSCLC adjuvant chemotherapy with CDDP and PEM versus CDDP and VNR (JIPANG study).^16^ The rate of treatment completion was significantly higher in the PEM arm (87.9% vs. 72.7%). Grade 3–5 events, especially hematological adverse events and febrile neutropenia occurred more often in the VNR arm (47.4% vs. 89.4%). As these results and the present study show, PEM is well tolerated and make the completion of four courses possible.

We also evaluated the short hydration method for CDDP administration in the postoperative setting. The conventional method involves large‐volume replacement of 3 L or more over 10 hours to avoid nephrotoxicity due to CDDP. On the other hand, short hydration methods are performed to achieve rehydration with approximately 2 L in four hours. Several studies have reported the safety of short hydration by combining proper magnesium supplementation with timely forced diuresis.^12^, ^17^, ^18^, ^19^ In addition, the development and implementation of antiemetic therapies, such as serotonin antagonists and neurokinin 1 receptor inhibitors, impressively improved cisplatin‐induced nausea and vomiting.^20^, ^21^This improvement of antiemetic therapies plays an essential role to complete treatment. This can be inferred from the difference between the completion rate in the past (about 50%) and that in VNR arm in the JIPANG study (72.7%).^3^, ^4^, ^5^, ^7^, ^16^ The combination of advanced antiemetic therapy and a short hydration method enables outpatient treatment to maintain patient QOL. For patients with a good PS who undergo surgery, outpatient‐based treatment is desirable for postoperative treatment. In the present study, the rate of outpatient chemotherapy completion after the second cycle was over 90%. Thus, we showed the feasibility of CDDP and PEM adjuvant therapy with a short hydration method for NSCLC patients after lobectomy.

The two‐year RFS in this study was similar to that recorded in other CDDP‐based adjuvant chemotherapy studies.^3^, ^4^, ^5^, ^7^ In the JIPANG study, the three‐year RFS of PEM arm was similar to that of VNR arm.^16^ If CDDP and PEM become available as a postsurgical treatment, the present study could be referred to as an outpatient‐based treatment.

The present study had some limitations. First, the sample size was small, but sufficient for assessing the feasibility of the adjuvant chemotherapy regimen. Second, since the present study was not a comparative design, we could not compare that of other regimens. Third, research was planned to be conducted at multiple facilities, but only two facilities registered. The JIPANG study seems to have solved these limitations.

In conclusion, this study indicates that adjuvant treatment with CDDP and PEM with a short hydration method is well tolerated for resected nonsquamous NSCLC patients with a good PS in the outpatient setting with limited toxicity. This regimen has the possibility to become one of the options of adjuvant therapy for these patients in the future.

## Disclosure

M. Tachihara reports lecture fees from Eli Lilly and Company. Y. Nishimura reports grants from Eli Lilly Japan K.K. There is no conflict of interest for the other coauthors.
